# Developmental tuning of mineralization drives morphological diversity of gill cover bones in sculpins and their relatives

**DOI:** 10.1002/evl3.128

**Published:** 2019-07-16

**Authors:** Eli G. Cytrynbaum, Clayton M. Small, Ronald Y. Kwon, Boaz Hung, Danny Kent, Yi‐Lin Yan, Matthew L. Knope, Ruth A. Bremiller, Thomas Desvignes, Charles B. Kimmel

**Affiliations:** ^1^ Institute of Neuroscience University of Oregon Eugene Oregon 97403; ^2^ Institute of Ecology and Evolution University of Oregon Eugene Oregon 97403; ^3^ Department of Orthopedics and Sports Medicine University of Washington Seattle Washington 98104; ^4^ Institute for Stem Cell and Regenerative Medicine University of Washington Seattle Washington 98104; ^5^ Department of Mechanical Engineering University of Washington Seattle Washington 98104; ^6^ Vancouver Aquarium Ocean Wise Vancouver BC V6G 3E2 Canada; ^7^ Department of Biology University of Hawai'i at Hilo Hilo Hawaii 96720

**Keywords:** Convergence, Cottoidei, evo‐devo, heterochrony, ossification, osteoid, radiation, Zoarcidae

## Abstract

The role of osteoblast placement in skeletal morphological variation is relatively well understood, but alternative developmental mechanisms affecting bone shape remain largely unknown. Specifically, very little attention has been paid to variation in later mineralization stages of intramembranous ossification as a driver of morphological diversity. We discover the occurrence of specific, sometimes large, regions of nonmineralized osteoid within bones that also contain mineralized tissue. We show through a variety of histological, molecular, and tomographic tests that this “extended” osteoid material is most likely nonmineralized bone matrix. This tissue type is a significant determinant of gill cover bone shape in the teleostean suborder Cottoidei. We demonstrate repeated evolution of extended osteoid in Cottoidei through ancestral state reconstruction and test for an association between extended osteoid variation and habitat differences among species. Through measurement of extended osteoid at various stages of gill cover development in species across the phylogeny, we gain insight into possible evolutionary developmental origins of the trait. We conclude that this fine‐tuned developmental regulation of bone matrix mineralization reflects heterochrony at multiple biological levels and is a novel mechanism for the evolution of diversity in skeletal morphology. This research lays the groundwork for a new model in which to study bone mineralization and evolutionary developmental processes, particularly as they may relate to adaptation during a prominent evolutionary radiation of fishes.

**Impact Summary**

We describe a new developmental trajectory for craniofacial bones in fishes based on histological, tomographic, and molecular evidence. The resulting tissue state, which we call “extended osteoid,” and the developmental down‐tuning of mineralization that presumably gives rise to it appear to be a heretofore undescribed phenomenon in evolutionary biology, and extended osteoid helps to explain a large proportion of the morphological diversity among opercle bones of bony fishes in the suborder Cottoidei. This membranous tissue in the opercle has likely evolved repeatedly in (and at least once outside of) cottoid fishes, although ecological bases for the pattern of convergence require future study. The repeated appearance of similar morphologies in distinct species offers evolutionary developmental biologists a unique opportunity to test which aspects of the genotype–phenotype map are repeatable on a macroevolutionary scale. Extended osteoid in the cottoids also holds promising potential as a model for studying osteogenesis, given biological separation of the processes of bone formation and its mineralization in a nonpathological context. We propose that variation in extended osteoid within and among various species of sculpin offers a potentially useful window into the genetics of mineralization‐related bone pathologies such as osteomalacia, and of repeated evolution of form via developmental processes in general.

Understanding the genetic, developmental, and evolutionary mechanisms that underlie morphological variation is an overarching aim in many branches of biology. Bone shape and size constitute fundamental components of morphological diversity, and a rich body of work using vertebrate animal models—primarily mouse and zebrafish—has identified developmental pathways that, when disrupted, significantly alter skeletal structure (see Clouthier and Schilling (2004) for a review of major craniofacial effectors). Large‐effect perturbations introduced in these studies affect processes such as pharyngeal arch patterning through Endothelin‐1‐dependent signaling cascades (Kurihara et al. 1994; Neuhauss et al. 1996; Ruest et al. 2003) and Krox‐20‐dependent skeleton‐wide ossification (Levi et al. 1996), so they often have severe fitness consequences and are unlikely to explain most of the variation we observe in nature. An important goal, therefore, is to identify developmental mechanisms that permit specific and less disruptive (but still morphologically significant) changes to bone shape relevant at the population and species levels. Such variants likely manifest during an extended period of development, resulting in a reshaping or resizing of bones we observe and recognize as homologous in different species. Crucial to the dynamic process of bone development are mesenchymal stem cell‐derived osteoblasts, cells responsible for the secretion and ultimately the mineralization of bone matrix. Our previous work in zebrafish, for example, revealed that modular regulation of osteoblast placement within a skeletogenic mesenchymal condensation is a critical determinant of bone shape (Kimmel et al. 2010). Another osteoblast‐specific mechanism of shape modification occurs by increasing the number of osteoblasts in a region on the edge of the bone, which can be accomplished by cell recruitment through fate switching (Takada et al. 2009), by cell migration (Fukuyama et al. 2004), or by local osteoblast or preosteoblast proliferation (Huycke et al. 2012; Kim et al. 2012).

Although the spatiotemporal distribution of osteoblasts is clearly an important determinant of bone shape, modification of later events during osteogenesis may require fewer perturbations to core regulatory networks important during skeletal development. A more modular “fine tuning” of late stages in bone development, therefore, might be especially accessible to evolutionary processes like natural selection (Wagner 1996; Brakefield 2006; Mengistu et al. 2016). During intramembranous ossification, demarcation of the bone tissue precursor is based on the spatial distribution of osteoblasts, but secretion of a collagen‐proteoglycan osteoid matrix must follow (Franz‐Odendaal et al. 2006). This osteoid tissue is transient, and provides the organic matrix for mineralized bone, in part through the expression of extracellular matrix proteins like Fibronectin, Type I Collagen, Bone Sialoprotein, Osteopontin, and Osteocalcin (Ducy et al. 1997; Cowles et al. 1998). It is entirely conceivable that a bone's effective shape, and ultimately its function, could be determined by the extent to which mineralization of the osteoid matrix occurs, serving as a potentially important mechanism for the developmental tuning of skeletal shape and ultimately the generation of variation at a macroevolutionary scale.

We have observed widespread variation across acanthomorph fishes in the shape of the mineralized opercle (OP), the primary gill cover supporting bone (Kimmel et al. 2017). Based on our study of OP morphospace, some of the most striking variation over a relatively short phylogenetic distance occurs in fishes of the suborder Cottoidei, which includes sculpins, sandfishes, and snailfishes (Smith and Busby 2014). In this article, we refer to all fish in the suborder Cottoidei as “cottoids.” To avoid confusion, when referring to the cottoid superfamily Cottoidea (Jordaniidae, Rhamphocottidae, Scorpaenichthyidae, Agonidae, Cottidae, and Psychrolutidae), we will use the full superfamily name. The Cottoidea, which include “sculpins” and “poachers,” underwent a morphologically diverse radiation of species as recently as the Miocene (David 1943) and have evolved to occupy a range of environments, including multiple transitions from subtidal marine to intertidal (Knope and Scales 2013) and freshwater (Knope 2013; Goto et al. 2014) habitats. Indeed, the rates of lineage and body size diversification for this group have been calculated to rank among the top 10% of actinopterygian families (Rabosky et al. 2013), a category that also includes cichlids, a canonical group for the study of rapid diversification and speciation (Salzburger et al. 2005).

We find that sculpin lineages differ from one another dramatically with respect to the presence of mineralized material in the central, distal region of the OP, and to a much lesser extent in regions of neighboring bones including the subopercle (SOP) and the interopercle (IOP). This variation is likely of functional relevance given the important roles of gill cover bones in respiration (Hughes 1960), mouth opening, and predator defense (Anker 1974), and given associations between variation in OP shape and ecological variables in Lake Tanganyikan cichlids (Wilson et al. 2015), ariid catfishes (Stange et al. 2016), threespine stickleback (Kimmel et al. 2008; Kimmel et al. 2012), and icefishes (Wilson et al. 2013). Granted the diverse habitats in which sculpins and their relatives evolved, it is possible that OP shape divergence occurred in association with major ecological shifts. Intertidal and some freshwater habitats can be oxygen poor relative to deeper water, requiring fundamentally different respiratory adaptations (Mandic et al. 2009) that could include changes in gill cover movement or sealing performance. Large fish predators may be differentially abundant depending on habitat type, possibly requiring craniofacial modifications for protection (Cowan 1970; Anker 1974). Dissolved mineral availability is also variable among aquatic habitat types and could play a role in the evolution of bone mineralization among fishes (Bell et al. 1993).

Here, we study this exceptional diversity in mineralized shape of the OP bone among cottoid fishes (and several outgroups) and describe a prominent feature we call “extended osteoid,” bone matrix that makes up large regions of certain bones and is dynamic, pliable, and calcium deficient in nature. We evaluate the developmental and evolutionary significance of extended osteoid by addressing several important questions about its properties and diversity: (1) Are the large, membranous regions of gill cover bones observed across species biologically classifiable as osteoid? (2) Is the majority of OP mineralized shape variation among species explained by extended osteoid? (3) Have largely nonmineralized OPs evolved multiple times across the cottoid phylogeny and beyond? And (4) How does the developmental timing of the appearance of extended osteoid vary among species? Although not a primary objective of our study, we also ask whether habitat characteristics such as gross depth and salinity are associated with osteoid‐related OP shape variables. In answering these questions, we propose the phenomenon of extended osteoid as a mechanism for explaining the major OP diversity of the Cottoidea and present a new vertebrate model to study the role of bone mineralization in development and evolution.

## Methods

### COLLECTION AND IDENTIFICATION OF SPECIMENS

The majority of individuals represented in this study were obtained as gifts or loans from other researchers, scientific collections, or aquaria. These include the Oregon State Ichthyology and Burke Museum collections, the Vancouver Aquarium, the Oregon Coast Aquarium, bycatch from other researchers at the University of Oregon, and fish from previous studies (Knope 2013; Knope and Scales 2013; Kimmel et al. 2017). All species considered in our current study, the number of individual specimens examined, the sources of the material, and the analyses based on them are included in Supporting Information File S1. Our survey included seven families from the suborder Cottoidei (Liparidae, Jordaniidae, Rhamphocottidae, Scorpaenichthyidae, Agonidae, Cottidae, and Psychrolutidae), sensu (Smith and Busby 2014), plus six outgroup perciform families (Hexagrammidae, Zoarcidae, Pholidae, Gasterosteidae, Sebastidae, and Centrarchidae).

We also collected some of our own specimens through hand netting and trapping in Oregon streams and tidepools. All fish taken live by the authors were captured, euthanized using a lethal dose of MS‐222 (tricaine), and preserved in ethanol or 4% paraformaldehyde (in situ hybridization samples only). This study was approved by the University of Oregon IACUC (Protocols #17–28 and #10–26), and all live animals used for this study were treated in accordance with these IACUC protocols.

Fish were morphologically keyed using Miller and Lea (1972) in addition to Markle et al. (1996). All initially ambiguous species designations were confirmed by BLAST searches based on cytochrome b mitochondrial DNA and s7 intron sequences, commonly used markers for understanding sculpin species relationships (Grachev et al. 1992; Ramon and Knope 2008). We extracted DNA from fin clips stored in 95% ethanol or stored at –80°C using a precipitation‐free, lysis‐only DNA extraction protocol (Westerfield 2000). Samples were PCR amplified for mitochondrial cytochrome b (GLUDG‐L: 5’‐TGACTTGAARAACAYCGTTG‐3’ and CB3‐H: 5’‐GGCAAATAGGAARTATCATTC‐3’ for marine species and L14724: 5’‐GTGACTTGAAAAACCACCGTT‐3’ and H15915: 5’‐CAACGATCCGGTTTACAAG‐3’ for *Cottus*) and the first intron of the nuclear S7 ribosomal protein (S72F: 5’‐TCTCAAGGCTCGGATACGTT‐3’ and S74R: 5’‐TACTGAACATGGCCGTTGTG‐3’ for all fish), followed by clean‐up and Sanger sequencing.

### SAMPLE PREPARATION FOR MORPHOLOGICAL ASSAY

#### Staining

To reveal patterns of craniofacial bone mineralization, we stained specimens using the acid‐free Alcian Blue and Alizarin Red double stain protocol (Walker and Kimmel 2007), which minimizes artifactual demineralization of bone. Alcian Blue stains cartilage and other tissues via binding to polysaccharides, and Alizarin Red stains mineralized bone tissue by binding directly with calcium‐containing compounds. Because we relied on specimens from a variety of sources, all fish used for morphological analysis had been preserved and stored conventionally, in 70% or 95% ethanol prior to staining. After staining, the bones were stored in 50% glycerol with 0.1% potassium hydroxide and a small amount of thymol.

#### Dissection

To quantify shape of individual bones, it was necessary to dissect and disarticulate each specimen. Using forceps, the dentaries were disarticulated along with the premaxillae and ceratohyals. The hyomandibula was disarticulated from the cranium, freeing the facial bones. The bones were then completely disarticulated and cleaned. This method was found to be most consistent while preserving all hard and soft tissues of bone elements. Very seldom, trypsin was used to help soften the tissue.

#### Imaging

To obtain high‐resolution images for both shape quantification of the OP and to gain insights into the compositional nature of bone tissues, dissected bones were flat mounted and imaged using bright‐field, Nomarski differential interference contrast and incident fluorescent lighting. Using Adobe Photoshop, we simplified the bones to detailed silhouettes, both including and excluding the extended osteoid portions. These silhouettes formed the basis of morphometric (shape) analyses and “proportion osteoid” calculations (see below), which were carried out using pixel counts.

### COMPOSITIONAL ANALYSIS OF EXTENDED OSTEOID

#### In situ hybridization

To visualize spatial patterns of gene expression in the OP for a known osteoblast‐diagnostic mRNA, we performed RNA in situ hybridization targeting the transcription factor *sp7* (also known as *osterix*). We designed the oligonucleotide probes using a conserved region in an alignment of coding sequences (Supporting Information File S2) from the assembled whole‐body transcriptomes of *Cottus perplexus*, *Oligocottus maculosus*, and *Clinocottus globiceps*. These transcriptome assemblies were generated with 100 nt paired‐end Illumina reads using Trinity (Grabherr et al. 2011), and they are part of a separate study. Probe design followed the methods of Albertson et al. (2010). In zebrafish, *sp7* is expressed robustly in osteoblasts and shows stronger specificity than other osteoblast‐expressed genes such as *runx2a* and *runx2b* (Li et al. 2009), justifying its use as a reliable marker for bone tissue from the onset of mineralization.

Upon euthanasia, heads of subadult *O. maculosus* and *C. globiceps* specimens were fixed in 4% PFA and incubated for ∼24 hours at 4°C, after which they were washed twice in PBT (1× PBS, 0.2% Tween‐20). Each head was then soaked in 1 mL for 3 minutes each in a progression of 25% methanol: 75% PBT; 50% methanol: 50% PBT; and 75% methanol: 25% PBT before soaking twice in 100% methanol. Heads were stored at –20°C in 100% methanol. We then dissected out the portion of the operculum containing the OP and SOP and performed in situ hybridization as described in (Yan et al. 2005).

#### Histology

To confirm that certain regions of the OP and SOP were indeed exclusively composed of either mineralized bone or osteoid, we used the sensitive tetrachrome staining procedure described by Ralis and Watkins (1992). We fixed heads of juvenile *C. perplexus*, and two eelpouts (*Ophthalmolycus amberensis* and *Lycenchelys tristichodon*) in 4% PFA, decalcified, embedded in paraffin, cut 10 µm cross sections, slide‐mounted these sections, and completed the staining procedure.

#### MicroCT

As an alternative approach to visualizing and understanding mineralization patterns in whole bones, we performed microCT scanning using a vivaCT40 (Scanco Medical, Switzerland). The OP from *Scorpaenichthys marmoratus* was scanned using the following settings: 38 µm isotropic voxel size, 55 kVp, 145 mA, 1024 samples, 500 proj/180°, and 200 ms integration time. High‐resolution scans of the OP from *O. maculosus* were acquired using the following settings: 10.5 µm isotropic voxel size, 55 kVp, 145 mA, 2048 samples, 1000 proj/180°, and 200 ms integration time. DICOM files of individual fish were generated using Scanco software and analyzed using FIJI.

### MORPHOMETRIC ANALYSIS OF OP SHAPE

To quantify OP shape variation, we used the extended eigenshape method of shape analysis, developed by MacLeod (1999); (MacLeod 2012), who kindly contributed software in the form of unpublished Mathematica notebooks that included all of the required procedures. The analysis is two dimensional, appropriate for the flattened form of the OP. We initially examined a set of 122 samples spanning 13 perciform families, which included some species replicates and juvenile and subadult stages. For the primary analyses in the current study, however, we culled this set to include only adults, and in the interest of equal taxon sampling and limited specimen availability, only a single individual per species. We investigated the validity of this approach via a small within‐species analysis (see below). Using this culled set of 43 species, we placed landmarks at three specific regions of the bone edge: (1) at the middle of the joint socket for the articulation the OP makes with the hyomandibula, (2) at the tip of the ventral spur, and (3) at the tip of the posterior spur. These landmarks separated three segments along the bone outline that we demarcated using semi‐landmarks, placed at equal intervals to one another along the mineralized portion of the bone edge, to examine shape variation within these segments: (1) 11 semi‐landmarks along the “anterior segment” between landmarks 1 and 2; (2) 57 semi‐landmarks along the “ventral‐posterior segment” between landmarks 2 and 3; and (3) 19 semi‐landmarks along the “dorsal‐posterior segment” between landmarks 3 and 1. These *xy* coordinates (90 in all) captured >90% of shape variation of the OP perimeter. The coordinates were Procrustes transformed to reduce variation due to size and orientation, and the resulting shape coordinates subjected to principal component analysis (PCA). Because Procrustes transformation removes isometric, but not allometric, effects of size on shape (Outomuro and Johansson 2017), we included size as a covariate in phylogenetic generalized least squares (PGLS) analyses (see below). Size did not covary significantly with shape variables after accounting for phylogeny (see Results), so we did not apply any size‐based scaling factors to shape variables in subsequent analyses. We chose standard PCA, as opposed to phylogenetic PCA (pPCA; Revell 2009), to identify and characterize the principal shape changes contributing to morphological diversity, because leading pPCA axes may not adequately capture morphological divergence that occurs at internal nodes, and PCA does not require independence of variables like other comparative analyses (Polly et al. 2013).

To evaluate confidence in precisely capturing among‐species OP shape variation given just one specimen per species, we also performed PCA as above using a small set of seven species (see Supporting Information File S1) for which at least two individual replicates per species were available. We quantified species‐level repeatability and 95% confidence intervals for each principal component (PC) using the repeated measures framework described by Lessells and Boag (1987), as implemented by the R package rptR (Stoffel et al. 2017).

### PHYLOGENETIC COMPARATIVE ANALYSES OF OP SHAPE AND EXTENDED OSTEOID

To better understand evolutionary transitions in OP shape and extended osteoid, we conducted a series of phylogenetic comparative analyses based on a recently published, time‐calibrated tree for 11,638 actinopterygian fish species (Rabosky et al. 2018). From this large tree, we extracted a subtree for 42 of the 43 species featured in our extended eigenshape analysis (see above). One species (*Porocottus camtschaticus*) was not represented in the Rabosky et al. tree, so we added *P. camtschaticus* to our subtree using the Rabosky et al. divergence time estimate for *Gymnocanthus galeatus*, which belongs to the sister genus of *Porocottus*, according to Smith and Busby (2014).

We evaluated all‐rates‐equal (ER) and all‐rates‐different (ARD) models of evolution for the binary shape classification of “fan or fork” using the *ace* function from the R package ape (Paradis et al. 2004). For PC1 and the proportion of the OP composed of extended osteoid, we used the *fitContinuous* function of the R package GEIGER (Harmon et al. 2008) to first evaluate four models of continuous character evolution: no phylogenetic signal (*λ* = 0), Brownian motion (BM), Ornstein–Uhlenbeck (OU), and “early burst” (EB). An absence of phylogenetic signal would indicate no relationship between osteoid‐based OP shape divergence and evolutionary distance among lineages, whereas BM assumes a constant rate of divergence in shape from an initial state over evolutionary time and would be expected to produce phylogenetic signal without constraint. OU includes additional parameters that describe a trait optimum and constraining forces that reduce divergence from the optimum over evolutionary time. An EB model would suggest a decreasing rate of osteoid‐based OP change, with the most rapid divergence occurring at the base of the tree and consistent with adaptive radiation. Assuming no fundamental constraint on osteoid‐related OP shape and no clear expectation for a radiation from the base of tree included here, BM should fit the data better than the other models. We conducted all comparative analyses using version 3.3.2 of the R statistical language (R Core Team 2016).

#### PGLS analysis

To initially establish whether PC1 might serve as a reliable indicator for the proportion of OP area composed of extended osteoid, we plotted the relationship between the two variables and assessed the significance of this relationship by comparing full and reduced PGLS models via likelihood ratio test. Given the strength of this relationship (see section “Results”), further phylogenetic comparative analyses focused on PC1 as the dependent variable.

To evaluate the potential for associations between OP shape and habitat, while accounting for phylogeny and OP size, we fit a series of PGLS models to the data, excluding the phylogenetically distant and derived freshwater outgroup Centrarchidae. We estimated the phylogenetic correlation matrix under BM, Pagel's lambda, and OU evolutionary models using the *corBrownian*, *corPagel*, and *corMartins* functions of ape, respectively, which we then used to fit *gls* linear models describing the relationship between trait values and environment. Habitat designations for species, tested separately as a two‐level factor (deep/shallow) and a three‐level factor (deep marine/shallow marine/freshwater or estuary), were based on data from fishbase.org and Knope and Scales (2013). Intertidal and “transitional” marine habitats and freshwater habitats were considered “shallow,” and subtidal marine habitats were considered “deep.” OP size, measured as the square root of OP area, was included as a covariate (see above). PGLS models were fit using the function *gls* from the R package nlme (Pinheiro 2017).

#### Ancestral state reconstruction

To better understand historical transitions in OP shape during evolution of the Cottoidei, we estimated ancestral OP states. We first performed binary (“fork” or “fan”) ancestral state reconstruction (ASR) for OP shape among adult fish from the 43 species featured in the extended eigenshape analysis (see above), using maximum likelihood and parsimony approaches. We used the *ace* function to infer discrete ancestral states using an ER model to calculate marginal likelihoods. Parsimony‐based inference was carried out using the *MPR* function of ape, in accordance with the approach of Narushima and Hanazawa (1997).

Using all of the PCA species except two for which we did not obtain a reliable estimate of the proportion of OP composed of osteoid (*C. globiceps* and *Enophrys bison*), we performed continuous‐character ASR and plotted change inferred from these states along branches in the phylogeny according to a color scale. Finally, we performed ASR for PC1 scores, generated 95% confidence intervals for each ancestral state point estimate, and used these CI boundaries to reproduce wireframe renderings for selected nodes in the tree. These analyses were carried out using the *fastAnc* function of the R package phytools (Revell 2012), assuming a BM model of character evolution.

#### Analysis of OP shape convergence using *convevol*

To more formally evaluate the evidence for multiple transitions from an ancestral, fan‐like OP devoid of extended osteoid to a fork‐like OP with extended osteoid, we tested the significance of two different convergence metrics proposed by Stayton (2015), using the designated R package *convevol*. Both metrics must be evaluated in a phylomorphospace framework (Sidlauskas 2008) and require a priori definition of the species that delineate the suspected morphospace region of convergence. The first metric, “C_1_,” measures the proportion of the maximum distance—between any two nodes among the focal species and their ancestors—“closed by subsequent evolution” and reflected by average similarity among the extant focal species (Stayton 2015). The second metric, “C_5_,” is simply the number of instances in which lineages pass into the zone of convergence from outside of it. We ran *convevol* based on the phylomorphospace of PC1 and PC2 from the eigenshape analysis described above, and we delineated the suspected convergence region by those 19 species having unambiguously fork‐like OPs with extended osteoid. We also used *convevol* to evaluate whether the observed C_1_ and C_5_ values were greater than expected under a BM model of phenotypic divergence by comparing them to distributions obtained from 999 evolutionary simulations.

## Results

### EXTENDED OSTEOID IS A PROMINENT FEATURE OF COTTOID OPERCULAR BONES

#### Extended osteoid is common in cottoid fishes

**Figure 1:**
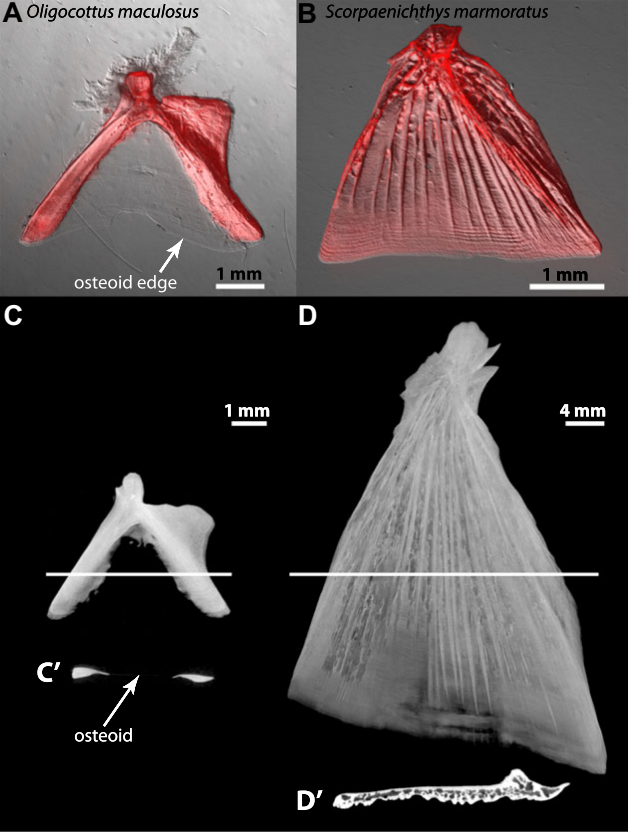
Extended osteoid shows a lack of calcification consistent with known nonmineralized tissues. (A) An adult *Oligocottus maculosus* OP stained with Alizarin Red, showing lack of stain in the region of extended osteoid. (B) A juvenile *Scorpaenichthys marmoratus* OP stained with Alizarin Red, showing full calcification throughout the bone. (C) An adult *O. maculosus* OP scanned using microcomputed tomography (microCT) clearly shows mineralized and nonmineralized (extended osteoid) regions, and a computed cross section of the scan (C’) shows a nearly undetectable tissue layer in the extended osteoid region (arrow). (D) An adult *S. marmoratus* OP scanned using microCT clearly shows consistent mineralization throughout, albeit with heterogeneous density typical of reticular bone, as seen in the virtual cross section (D’).

Eighteen of the 19 genera we surveyed within the Cottoidei exhibited at least some flexible, membranous tissue within the three primary bones that make up the operculum or “gill cover.” This membrane appeared to lack calcification, as indicated by clear regions negative for Alizarin Red staining (Fig. 1A and B; Fig. S1). We named the membrane “extended osteoid” to reflect its likely makeup as a long lasting, dynamically regulated osteoid matrix that appears to undergo a delay in the final stages of intramembranous ossification (Cowles et al. 1998). With the possible exception of Agonidae (poachers), all cottoids we surveyed possessed this extended osteoid as part of the SOP, where it lines the ventral and to a lesser extent dorsal edges of the SOP's posterior projection. Additionally, some cottoid species demonstrated osteoid membrane located at the ventral edge of the IOP. We observed a remarkable degree of variation across the superfamily Cottoidea in the amount of extended osteoid present in the OP, ranging from a complete or nearly complete absence of this tissue type (a fully Alizarin Red‐stained “fan” shape) to a “fork” shape in which the entire central portion of the OP was unstained (Fig. 1A and B, respectively). We also surveyed nine (noncottoid) outgroup genera from the order Perciformes (Betancur‐R et al. 2017), representing the families Hexagrammidae, Zoarcidae, Pholidae, Gasterosteidae, Sebastidae, and Centrarchidae. Of these outgroups, we found evidence for noncalcified, membranous bone only in the OPs of the two zoarcids we examined, the eelpout species *L. tristichodon* and *O. amberensis* (Fig. S2A–E).

#### Extended osteoid is fundamentally distinct from mineralized bone

Alizarin Red staining revealed that although calcified portions of bones stained robustly, extended osteoid regions did not take up stain noticeably more than the background levels present in other tissue types known to be nonmineralized, such as connective tissue (Fig. 1A). Furthermore, microcomputed tomography (microCT) scanning of an *O. maculosus* OP, for example, showed the extended osteoid to display extremely low radiopacity (Fig. 1C), as confirmed by an image constructed perpendicular to the plane of the bone (Fig. 1C′), and suggesting the presence of very thin, likely nonmineralized tissue between the two heavily mineralized “struts.” Conversely, the species *S. marmoratus* (a more basal lineage in Superfamily Cottoidea) showed little, if any, OP extended osteoid. We observed positive Alizarin Red staining throughout most of its OP, except for a very thin strip of about 30 µm at the leading edge (Fig. 1B). A (microCT) scan for a *S. marmoratus* OP also suggested clear mineralization throughout most of the bone (Fig. 1D–D′).

**Figure 2:**
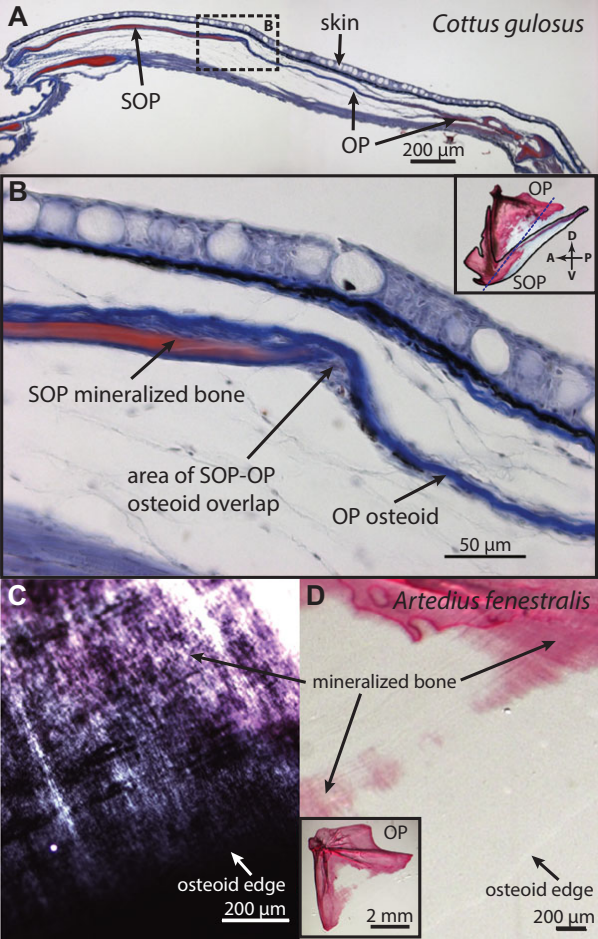
Ralis–Watkins staining and “growth bands” confirm the osteoid nature of the nonmineralized membrane in OPs of two “fork‐bearing” sculpins. (A) A cross section of a Ralis–Watkins stained *Cottus gulosus* gill cover, with the mineralized portion of the OP and SOP in red and the extended osteoid in blue, showing a lack of mineralization in the extended osteoid region. The two OP arrows straddle the transition between nonmineralized and mineralized regions of the OP. (B) Higher magnification of the same image, corresponding to the dashed rectangle in (A). The inset shows the orientation of the cross section (dashed line) with respect to the OP and SOP, drawn on an Alizarin‐stained preparation from a different *C*. *gulosus* individual. (C) Alizarin‐stained *Artedius fenestralis* OP showing banding continuous between the mineralized (pink) and nonmineralized (dark gray) portions of the bone, visualized with Nomarski differential interference contrast microscopy. (D) Same image with lower magnification and cross‐polarized light. Inset is a view of the entire bone.

Ralis–Watkins staining, which is a standard method for differentiating mineralized bone from osteoid (Ralis and Watkins 1992), confirmed that in *Cottus gulosus* (Fig. 2A and B), and two noncottoid (eelpout) species (Fig. S2C and D′), extended osteoid was not simply extremely thin, canonically mineralized bone. The extended osteoid stained a deep blue, as one expects for osteoid, while the surrounding mineralized bone stained red, as one expects for mineralized bone.

#### Structural and cellular evidence supports extended osteoid as true osteoid

Nomarski and cross polarized light imaging revealed a developmental signature of incremental banding (“growth rings”) in extended osteoid regions of the OP, which was in phase with banding of the adjacent mineralized bone (Fig. 2C and D). This pattern is consistent with an ordered structure of premineralized bone matrix (Reznikov et al. 2018) and known circadian “periodicity” in osteoblast proliferation (Fu et al. 2005), and therefore is an expectation for osteoid independent of the mineralization process. The banding we observed was synchronized and continuous between mineralized and nonmineralized regions of the OP (Fig. 2D), suggesting that extended osteoid initially forms in conjunction with the neighboring osteoid that ultimately follows the more standard mineralization trajectory.

In situ hybridization in juvenile *O. maculosus* and *Clinocottus globiceps* revealed robust transcription of *sp7* at the growing edge of OP extended osteoid (Fig. S3). *sp7* is expressed by differentiating osteoblasts with high specificity at the osteogenic front of the OP in zebrafish (Li et al. 2009; Huycke et al. 2012). The lining of extended osteoid with functional osteoblasts further suggests that this tissue is indeed a form of osteoid.

### EXTENDED OSTEOID EXPLAINS THE MAJOR AXIS OF OP SHAPE VARIATION IN COTTOID FISHES AND BEYOND

**Figure 3:**
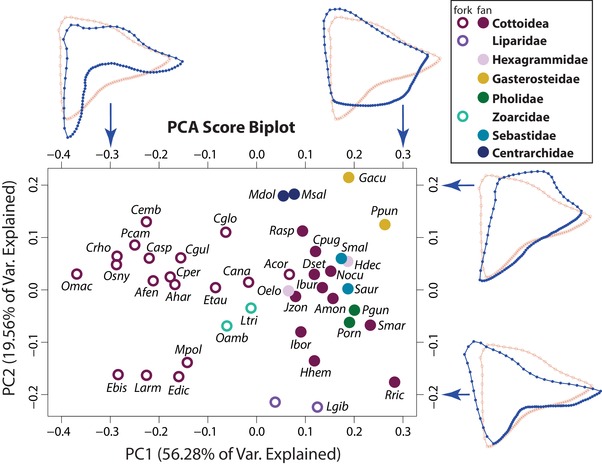
The “fan‐to‐fork” continuum, driven largely by extended osteoid, is a major component of OP shape variation among cottoid fishes and their relatives. An eigenshape‐based PCA morphospace (PC1 and PC2) of OP shapes only taking mineralized bone into account. Species are coded as either forks (open circles) or fans (closed circles) and colored by taxonomy. Wireframe reconstructions for two extreme values (in blue) along both PCs are included to illustrate shape variation. The superimposed red wireframe reconstruction corresponds to shape at PC1 = 0 and PC2 = 0. Note that the major axis of shape variation (PC1) separates fans and forks quite well, also indicated by the blue PC1 wireframe reconstructions. PC2, on the other hand, summarizes other attributes of shape.

As mentioned, species from the superfamily Cottoidea (Smith and Busby 2014) fell along a continuum from fan‐shaped to fork‐shaped OPs, when considering the calcified (Alizarin Red‐stained) bone outline (Fig. S4). We performed a shape‐based PCA including this monophyletic group and seven additional families, using only mineralized OP outlines (excluding extended osteoid) to define “effective” shape. PC1 of mineralized OP PCA explained 56.28% of the total shape variation, and it mostly separated “fan‐like” from “fork‐like” shapes (Fig. 3). PC2, which explained 19.56% of the total shape variation, was associated with the presence of a protruding dorsal‐posterior OP edge, a location of attachment for the levator operculi muscle (Yabe 1985), suggesting important functional shape variation independent of extended osteoid and the fan‐fork continuum.

Because the above analysis was based on a single specimen per species, we performed an additional PCA using seven species for which at least two individuals were included to ensure that intraspecific variation was not so high as to call into question our interpretations of among‐species variation. Based on this smaller analysis, within‐species variation was much lower, on average, than among‐species variation in the space defined by the first two principal components (Fig. S5A). Repeatability at the species level was extremely high for the first two principal components (PC1: 0.99 [0.95, 1.0]; PC2: 0.96 [0.79, 0.99], and significantly greater than 0 for the first six PCs; Fig. S5B).

PC1 values from the 43‐species analysis were tightly (negatively) associated with the proportion of OP area occupied by extended osteoid (Fig. S6C), and this relationship was significant after accounting for phylogeny using PGLS (*b* = –1.00 [–1.13, –0.87]; LRT: $\chi_{1}^{2}$ = 79.57; *P* < 0.0001). These insights imply that the presence of extended osteoid is a major feature underlying OP shape distinctions of some cottoid species and their outgroups (i.e., zoarcids), and that extended osteoid is a primary driver of bone shape likely to influence functional variation of the gill cover.

### EVIDENCE FOR A SUBTLE ASSOCIATION BETWEEN OP SHAPE VARIATION AND HABITAT

Using PGLS we tested whether PC1 was associated with habitat attributes. When habitat was treated as a two‐level factor (“shallow” vs. “deep”), we noted a small but significant effect: a mean reduction of 0.13 PC1 units in shallow—relative to deep—dwelling species (Table 1; LRT: $\chi_{1}^{2}$ = 4.29; *P* = 0.038). We noted a similar effect size (a mean increase of 0.12 in shallow species) when considering the proportion of OP composed of extended osteoid as the response variable (Supporting Information File S3; LRT: $\chi_{1}^{2}$ = 4.71; *P* = 0.030). When habitat was treated as a three‐level factor (“freshwater/estuary” vs. “shallow marine” vs. “deep marine”), the significance of its effect on PC1 was less apparent via Akaike Information Criterion (AICc) and likelihood ratio test (Table 1; LRT: $\chi_{1}^{2}$ = 4.80; *P* = 0.091), most likely owing to reduced statistical power via fewer observations per factor level. PGLS models assuming a BM model of trait evolution performed best in all cases (Supporting Information File S3). Finally, it should be noted that in all cases dropping the OP size covariate from the full model did not worsen the fit, suggesting no systematic relationship between OP shape and size after accounting for phylogeny (Table 1; Supporting Information File S3).

**Table 1:** The major axis of OP shape (PC1) is subtly associated with basic environmental categories reflecting habitat depth (2‐level factor) and habitat depth/type (3‐level factor)

PGLS model	AICc	dAICc	Log likelihood	LRT statistic	LRT d.f.	LRT *P*
Full: PC1 ∼ Habitat (2) + OPsize	−40.52	1.94	24.82			
No Habitat: PC1 ∼ OPsize	−38.69	3.77	22.67	42.29	1	0.038
No OPsize: PC1 ∼ Habitat (2)	−42.47	0	24.56	0.52	1	0.47
Full: PC1 ∼ Habitat (3) + OPsize	−38.43	2.13	25.07			
No Habitat: PC1 ∼ OPsize	−38.69	1.86	22.67	4.80	2	0.091
No OPsize: PC1 ∼ Habitat (3)	−40.55	0	24.83	0.48	1	0.49

Shown are results from phylogenetic generalized least squares (PGLS) hypothesis tests in which phylogeny was accounted for and assuming a BM model of trait evolution. In the two‐level analysis, environmental factor levels were “deep” and “shallow.” In the three‐level analysis, environmental factors levels were “deep marine,” “shallow marine,” and “estuary/freshwater.” AICc, Akaike Information Criterion corrected for small sample sizes; dAICc, AICc – minimum AICc; LRT, Likelihood Ratio Test; d.f., degrees of freedom. In both cases, the PGLS model including habitat, but excluding OP size, was the best fit.

### EXTENDED OSTEOID AND FORK‐SHAPED OPs HAVE EVOLVED MULTIPLE TIMES

#### Evaluation of evolutionary models

To facilitate ASR, we first compared models of OP attribute evolution using likelihood ratio tests and an information theoretic approach. We determined that the ARD model of evolution for the binary trait of “fan or fork” yielded no better a fit to the data than the ER model (LRT: $\chi_{1}^{2}$ = 1.45; *P* = 0.23), so ER was assumed when reconstructing ancestral character states. We differentiated the fits of no phylogenetic signal, BM, OU, and EB models for both PC1 and proportion of OP area composed of osteoid, based on second‐order corrected Akaike Information Criterion (AICc) differences of 2 or greater (Burnham and Anderson 2007). For both OP variables, we found comparably good fits of BM and OU models, and very low support for no phylogenetic signal and EB models (Supporting Information File S4). Given the similar support for BM and OU, we assumed BM, the model with fewer parameters, for the continuous trait ASRs.

#### Ancestral state reconstruction

**Figure 4:**
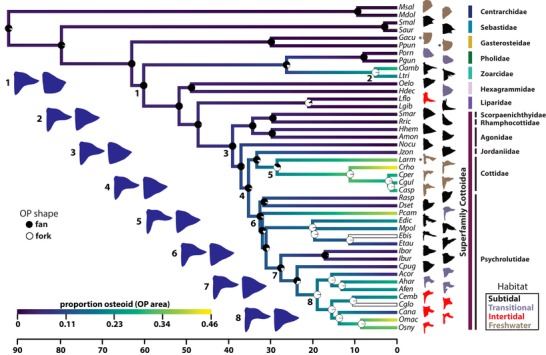
A rich diversity of OP shape and multiple appearances of extended osteoid have occurred during the evolution of cottoid fishes and their relatives. Depicted are likelihood‐based ancestral state reconstructions for three OP shape traits: fan/fork, PC1, and proportion of the OP composed of extended osteoid. Circles at internal nodes convey marginal likelihoods for either fan or fork state via black and white areas, respectively, within each circle. Lower (left) and upper (right) 95% confidence limits for PC1 ancestral estimates are depicted by blue, filled wireframe drawings at selected, numbered nodes. Ancestral reconstructions for proportion of OP composed of osteoid are depicted via a color gradient applied to branches in the time‐calibrated phylogeny. Terminal branches for *Ebis* and *Cglo* are white, because the proportion osteoid measurements were not reliable for these species. The branch length scale is in units of millions of years. Species silhouettes are outlines of mineralized (Alizarin‐positive) regions from actual bone photographs, although not to scale. Families are colored (vertical bars) according to the scheme from Fig. 3. OP silhouette colors correspond to the four main habitats these fish occupy in nature. Species labeled with an asterisk are found in marine or estuarine habitats in addition to freshwater. The four lineages with especially fork‐like OPs include Zoarcids (*Oamb*; *Ltri*), Cottids (*Larm*; *Crho*; *Cper*; *Cgul*; *Casp*), *Porocottus* (*Pcam*), and *Oligocottus* (*Omac*; *Osny*), and this shape appears to be derived independently in each lineage.

Based on binary ASR using both maximum likelihood and parsimony approaches, a fan‐shaped OP is most likely ancestral with respect to the Cottoidei, and a fork‐shaped OP has likely arisen 3–4 times within cottoids (Fig. 4; Fig. S6A). The snailfishes (family Liparidae) do demonstrate modest fork‐shaped OPs, but there is no evidence for extended osteoid in the center of the bone in this lineage, and they do not fit cleanly into the fan‐fork dichotomy. Rather their OPs are “cowboy boot shaped” (Fig. 4), with the notch adjacent to the “boot heel” devoid of extended osteoid. We classified them as forks for the purpose of the ASR but note that the two species we studied (*Liparis gibbus* and *Liparis florae*) occupied a region of PC1 near species with fan‐shaped OPs. We also found evidence for derived fork‐like OPs outside of the Cottoidei, in two species of eelpout (family Zoarcidae). The eelpouts we examined (*O. amberensis* and *L. tristichodon*) also demonstrated nonmineralized OP membrane tissue (Fig. S2A, B, and E), suggesting that osteoid‐associated, fork‐like OP variants are likely not restricted to cottoid fishes.

Maximum likelihood ASR treating PC1 and the proportion of bone made up of extended osteoid as continuous traits also revealed multiple evolutionary events of OP mineralization down‐tuning (Fig. 4). Here, we inferred clear cases of large increase in extended osteoid (and therefore decrease in PC1 scores) for eelpouts, the lineage leading to sculpin genera *Leptocottus* and *Cottus*, the lineage leading to the sculpin genus *Porocottus*, and the tidepool‐dwelling sculpin clade, with extreme examples in genus *Oligocottus* (Fig. 4).

#### Analysis of evolutionary convergence using *convevol*

We used the R package *convevol* to estimate two morphospace metrics of convergence, one distance based (C_1_) and one event‐frequency based (C_5_). In the phylomorphospace defined by PCs 1 and 2, we observed a C_1_ estimate of 0.33, which was significantly greater than expected, based on 999 evolutionary simulations under BM (*P* = 0.001). We observed a C_5_ value of 3 independent transits into our defined region of suspected convergence, all transits occurring mostly along PC1 in the direction of increasing “forkiness” (Fig. S6B). This frequency was not unexpectedly high relative to BM simulations (*P* = 0.56). Although evidence for especially high phenotypic similarity among extant taxa relative to the maximal distance between ancestral lineages was strong, the sheer number of convergent “events” was not exceptionally high.

### EXTENDED OSTEOID APPEARS LATE IN DEVELOPMENT

**Figure 5:**
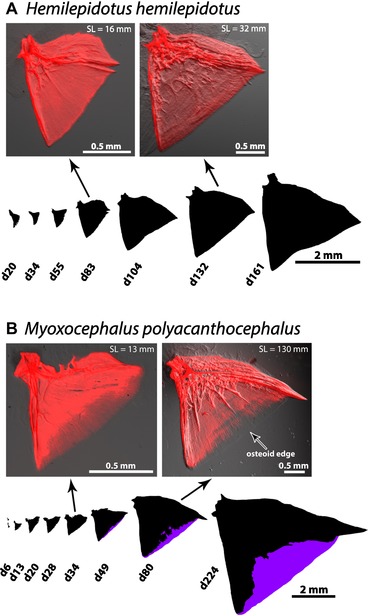
Extended osteoid and the resulting “fork” morphology appear after an established “fan” shape, relatively late in OP development. (A) A developmental series from a species (*H*. *hemilepidotus*) with a stereotypical, ancestral fan OP, depicted by mineralized bone outlines and Alizarin Red images for two of the outlines. (B) A similar series for the fork species *M. polyacanthocephalus*. Extended osteoid in *M. polyacanthocephalus* does not appear until 49 days posthatching, and the proportion of the total bone area it occupies increases thereafter. The edge of extended osteoid, which is difficult to see owing to the transparency of osteoid, is marked with an arrow.

How might development have evolved to express extended osteoid and the distinctive fork morphology in the superfamily Cottoidea? One possibility is that bone patterning is reconfigured from the earliest stages of bone ontogeny. On the contrary, our observations of OP form throughout bone development in multiple species revealed that all surveyed OPs began as fans regardless of whether the adult displayed fork or fan shapes, and that large regions of extended osteoid are observed late in bone formation (Fig. 5). In species with fan‐shaped OPs, fans were present throughout larval and juvenile stages, as illustrated by *Hemilepidotus hemilepidotus* in Fig. 5A. Four additional fan species spanning the Cottoidea (*Jordania zonope*, *Rhamphocottus richardsonii*, *Nauthichthys oculofasciatus*, and *Ruscarius meanyi*) also demonstrated this pattern. Conversely, in species with fork‐shaped OPs such as *Myoxocephalus polyacanthocephalus*, we did not observe any extended osteoid in the five young larval stages sampled (Fig. 5B). The first extended osteoid appeared by 49 days posthatching (d49), and the proportion of osteoid increased progressively (from 6% at d49 to 11% at d80 to 28% in the juvenile for the examples in Fig. 5B). The OP of the d224 subadult (last image in the sequence shown in Fig. 5B) is a well‐defined fork. Fan‐shaped OPs also preceded fork‐shaped OPs in other fork‐bearing species we examined at fewer developmental stages (*Artedius harringtoni*, *Clinocottus acuticeps*, and *O. maculosus*), representing multiple fan‐to‐fork transitions within the family Psychrolutidae. Furthermore, in the noncottoid eelpout species *O*. *amberensis*, we did not observe a fork‐like appearance until later stages of development (Fig. S2E). These observations suggest that the fork‐fan developmental dichotomy we observed is likely to be general within the superfamily Cottoidea, and very likely beyond, given a similar pattern in the outgroup family Zoarcidae.

### THE LOCATION OF EXTENDED OSTEOID CHANGES WITH BONE GROWTH: EXTENDED OSTEOID IS NOT PERMANENT OSTEOID

**Figure 6:**
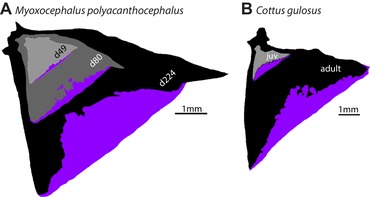
Extended osteoid is not permanent osteoid, but dynamically relocates during OP growth. Multiple stages of OP development for two fork species, superimposed at the joint in the bone outlines to “track” bone positions throughout growth. Clearly, extended osteoid during early stages of development after its appearance eventually becomes mineralized as the bone grows. For example, osteoid (purple) regions in early stages are mineralized (gray/black) regions in later stages. Extended osteoid patterns in *M. polyacanthocephalus* (A) and *Cottus gulosus* (B).

Once extended osteoid appears in development, is its position stable in the bone as the OP continues growth? We evaluated this question by analyzing overlays of the OPs of different stages, as shown in Figure 6, placing younger OPs on top of older ones. We placed the younger bones near the joint region of older ones (upper left in Fig. 6A and B), for this is the region where matrix outgrowth begins, as supported by analysis in zebrafish (Kimmel et al. 2010) and the orientation of the incremental banding patterns in sculpins (Fig. 2C and D) and other teleosts (Kimmel et al. 2005; Thuong et al. 2017). We observed outgrowth from the joint region toward the OP ventral‐posterior edge (lower right), the newest matrix to be formed (see section “Discussion”). For *M*. *polyacanthocephalus*, the earliest stage shown in Figure 6A is d49, which was the earliest we detected extended osteoid. This first extended osteoid was clearly present at the outgrowing edge, and its location clearly shifted to the edge of an OP from a later time point (d80), revealing dynamic regulation of osteoid position as the bone grows in a posterior‐ventral direction.

It was also evident from the overlays that regions of extended osteoid at young stages corresponded to regions of mineralized bone at the later stages. Extended osteoid replacement continued throughout growth; Figure 6B shows such replacement between a young juvenile and an adult of *Cottus gulosus*, an example from the family Cottidae. We note in this example that the OP forms spanning the juvenile–adult transition were quite similar to one another in spite of the marked outgrowth between the two stages, whereas the shape (and proportion of area) of the extended osteoid differed markedly across the larva–juvenile transition sampled in the *M*. *polyacanthocephalus* series (Fig. 6A).

## Discussion

### A NOVEL BONE DEVELOPMENT TRAJECTORY, WIDESPREAD AMONG COTTOID FISHES

We previously surveyed OP variation across 110 teleost families (Kimmel et al. 2017), but a subsequent, expanded focus on the diverse superfamily Cottoidea revealed substantial regions of mineral‐deficient, membrane‐like tissue to be an unusual feature of OPs in this group. Outside of cryptic graphical references to this tissue type in anatomical line drawings of some sculpin species (Yabe 1985), it had not been previously described. What, we asked, is the material nature of this tissue and its development? Our current study suggests that the flexible membrane filling the posterior ventral portion of fork‐shaped OPs is a form of osteoid we refer to as “extended osteoid,” owing to its extended but dynamic presence as nonmineralized matrix. These findings are supported by a variety of analyses, including calcification detection (Alizarin Red staining), material density examination (microCT), and histological staining (Ralis–Watkins histochemistry), which all showed that the membrane observed within the OPs of several species in Cottoidea has extremely low radiopacity and calcium content, and is therefore distinct from mineralized bone. The nearly undetectable intensity of Alizarin Red signal in extended osteoid regions was comparable to that in nonbony tissue regions, suggesting extremely low or no calcification. We rule out the possibility that the lack of strong Alizarin Red signal in extended osteoid could be related to the extreme thinness of the tissue, because mineralized regions of very young larval zebrafish (Kimmel et al. 2010) and sculpin OP regions both show significant Alizarin Red signal well before they are as thick as the unstained extended osteoid regions we observed. This lack of evidence for mineralization, in combination with osteoid‐positive Ralis–Watkins histochemistry data, strongly suggests that extended osteoid is not simply thin, mineralized bone.

Data from our cross‐polarized imaging and in situ hybridization show that the structure and gene expression patterns of extended osteoid warrant its classification as bone matrix. Specifically, the continuity of form between the membrane and mineralized bone within the OP rules out a nonosteogenic tissue. This in‐phase growth banding of mineralized bone and extended osteoid tissues (Fig. 2D) suggests a common developmental origin, and recent structural studies of developing bone help explain the ordered pattern. A nanometer‐level analysis of compact lamellar bone by Reznikov et al. (2018) revealed a defined, hierarchical structure of matrix composed of collagen helices organized into collagen microfibrils, which are further organized into fibril bundles, and ultimately lamellae. According to this model, the initial levels of bone matrix structure form independently of mineralization and would therefore produce an ordered structure for osteoid as well as compact bone. This insight, and demonstrations that circadian rhythms generate periodicity in osteoblast proliferation (Fu et al. 2005; Iimura et al. 2012) and fracture healing (Kunimoto et al. 2016), is consistent with our interpretation of banding patterns as evidence for true osteoid. Furthermore, the clear presence of *sp7*‐expressing osteoblasts at the growing edge of the OP extended osteoid (Fig. S3) argues for an osteogenic origin. We conclude that extended osteoid develops in conjunction with neighboring osteoid that ultimately follows the more standard mineralization trajectory, and we observe this pattern in multiple cottoid lineages that have evolved OP extended osteoid independently (Fig. S4). The developmental tuning that underlies the delayed mineralization of extended osteoid provides a mechanism for the impressive heterogeneity in OP morphology that has evolved in the Cottoidea (Figs. 4 and S4).

Osteoid is a well‐recognized stage in bone development but is usually temporary (Ducy et al. 1997; Cowles et al. 1998). Indeed, persistent forms of osteoid are known to exist, although generally only pathologically, as in osteomalacia, a multifactorial disease in which osteoid forms normally but fails to mineralize sufficiently (Feng et al. 2013), or due to dietary deficiencies such as in phosphorus‐deficient fish (Witten et al. 2016). While lasting, nonmineralized bone matrix has been identified in the fin spines of some blenniid fish species and in the maxilla of the blenniid *Neoclinus blanchardi*, neither of these traits have been thoroughly described, and this thicker, more rigid tissue suggests a different developmental identity than the extended osteoid we describe here (Hastings and Springer 1994). Apart from the aforementioned, the occurrence of nonpathological osteoid within bone structures also containing normally mineralized tissue is, to our knowledge, undescribed.

### EXTENDED OSTEOID IS A MAJOR DRIVER OF DIVERSITY IN COTTOID GILL COVER MORPHOLOGY

As extended eigenshape and subsequent PCAs demonstrate, fan‐ and fork‐shaped OPs are well segregated within the morphospace along the first principal component axis (Fig. 3). Furthermore, the proportion of the OP area made up of extended osteoid covaries strongly with PC1 (Fig. S6C). Indeed, PC1 explains over half of the overall OP shape variation, indicating that variation in extended osteoid among the sampled species forms a major basis for variation in OP shape. OP outline regions loading most heavily on PC1 were concentrated at the center of the OP ventral‐posterior margin (Fig. S6D), where most of the variation regarding extended osteoid resided. This result is significant given the functional importance of the OP as the largest skeletal constituent of the teleost gill cover. The extreme flexibility and thinness of extended osteoid make it morphologically distinct from normally mineralized bone, which suggests that such variation could influence the performance of the gill cover, perhaps in terms of respiration rate, water movement, feeding, gill desiccation prevention, or protection against predators.

It is also likely that OP shape attributes independent of extended osteoid contribute meaningfully to functional variation among species. Outline regions loading most heavily on PC2 and PC3 were concentrated at the dorsal‐posterior edge and joint of the OP (Fig. S6D), locations of attachment for the levator operculi and dilator operculi muscles, respectively (Yabe 1985). Future studies of functional morphology will be necessary to address how shape variation at these regions of the OP might affect performance and ultimately fitness in a number of environmental and behavioral contexts.

### THE REPEATED OCCURRENCE OF EXTENDED OSTEOID SUGGESTS PARALLEL EVOLUTION OF FORK‐SHAPED OPs

Both maximum likelihood and parsimony ASR suggest (1) that fan‐shaped OPs are ancestral with respect to the superfamily Cottoidea (Figs. 4 and S6A), (2) that fork‐shaped OPs have arisen multiple times throughout the phylogeny (Figs. 4 and S6A), and (3) that deeply forked OPs have appeared independently at least four times including the case in Zoarcidae (Fig. 4). Within family Psychrolutidae alone the likelihood‐based analysis supports three derived fork appearances, but an equally parsimonious alternative (assuming symmetric transition probabilities) is an initial fan‐to‐fork transition at the base of the psychrolutid clade that excludes *Radulinus* and *Dasycottus*, followed by a “reversal” to the fan shape in the ancestor of *Icelinus* and *Chitonotus* lineages, followed by a subsequent reappearance of fork‐like OPs in the subfamily Oligocottinae (Fig. 4). A larger phylogenetic sampling of cottoid species, especially from the sculpin family Psychrolutidae, would be required to more rigorously differentiate between these alternatives. Other examples of fork‐like OPs (Kimmel et al. 2017) exist outside of the species we examined for this study, for example, in batfishes (Ogcocephalidae), but seemingly without the presence of extended osteoid. Similarly, the cottoid family Liparidae (snailfishes) demonstrates fork‐shaped OPs with no extended osteoid (see section “Results”). In these cases, the developmental processes leading to major OP shape variation are likely very different from those driven by extended osteoid in cottoids and zoarcids. Our evidence suggests that the repeated evolution of fork‐shaped OPs in cottoids and zoarcids has occurred via a similar developmental process (extended osteoid) and is therefore an example of “parallel” evolution, at least in the broad sense. Whether the same developmental pathways and individual genes have been modified in parallel among these lineages remains an open but intriguing question.

Based on our survey, it is clear that a certain evolutionary lability exists for the severity of extended osteoid, and therefore fork‐shaped OPs, in the superfamily Cottoidea. Whether this lability is related to a simple and tunable genetic “switch,” repeatedly adjusted during the sculpin radiation by selective means or otherwise, is currently unknown. Future genomic investigations into the genetic basis of OP shape variation are promising and quite possible, as scale (prickle) number in *Cottus* sculpins has been tied to the *ectodysplasin* pathway using QTL mapping (Cheng et al. 2015). Ultimately functional testing, informed by genotype–phenotype association studies in sculpins and/or bone mineralization regulatory networks understood from animal models like zebrafish, will be required to test whether a specific regulatory switch or developmental pathway has been repeatedly modified to tune extended osteoid evolution across the group.

Past studies of sculpins have reported significant links between habitat and other traits such as scale number, body size, and body shape (Knope and Scales 2013; Buser et al. 2017). However, recent work on phenotypic evolution in the sculpin subfamily Oligocottinae has not provided strong evidence for relationships between craniofacial morphology and depth at which these species are found (Buser et al. 2018; Buser et al. 2017). We analyzed shape variation in a single bone, among a much larger phylogenetic group of cottoids, including eight families and several outgroups. Specifically, we tested whether osteoid‐based OP variation is associated habitat depth and/or habitat type (i.e., marine vs. freshwater) to better understand the potential for adaptive roles in cottoid OP shape evolution. Depth may influence factors such as oxygen availability, temperature, pressure, and predator regime, any of which could act as selective agents on variation in respiration performance or mechanical protection. In addition, ions essential for bone mineralization (e.g., calcium and phosphate) are less available in freshwater and estuarine relative to marine habitats, which could in principle lead to selection for reduced mineralization of bony tissues in mineral‐poor habitats (Bell et al. 1993, Marchinko and Schluter 2007). We found a statistically significant association between OP shape (PC1 and proportion osteoid) and whether species live in shallow or deep water (Table 1), with osteoid‐rich, fork‐shaped OPs more commonly observed in shallow‐dwelling species. For example, we observed an absence of fan‐shaped OPs in species living in freshwater, estuary, and intertidal habitats, although deep‐dwelling species demonstrate both fan‐ and fork‐like OPs (Fig. 4). These grossly defined deep and shallow habitat categories could covary with a number of more specific environmental variables, however, so interpretation of our initial results is not straightforward and should be exercised with caution. We also analyzed OP shape variation as a function of three habitat categories (“deep marine,” “shallow marine,” and “estuary/freshwater”). The importance of the habitat term in these PGLS models was less clear (Table 1), possibly due at least in part to a reduction in sample size within factor levels, relative to the above models with a two‐level habitat term.

It is possible that unknown attributes of habitat, life history, behavioral ecology, or otherwise—those not measured in our study—are fundamentally associated with variation in OP shape, and particularly the multiple fan–fork transitions we have identified. For instance, the extent to which the OP might integrate with other bones of the gill cover in cottoid‐specific functional capacities remains unexplored. The preopercle (POP), which lies anteriorly to the OP, is adorned with long, sharp spines in many cottoid lineages (see Fig. S1A and B). The POP can be extended by gill cover “flaring” behavior when threatened (Cowan 1969, Cowan 1970), but it is currently unclear how extended osteoid in the OP, SOP, and IOP might influence this trait, given that POP erection is controlled by a separate musculature (Cowan 1969). Many of these questions lie beyond the purview of the work presented here, and future phylogenetic comparative studies leveraging greater taxonomic sampling, functional morphology, and more detailed species‐specific data on diverse features of cottoid biology are clearly warranted.

Regardless of the possible ecological underpinnings, our work suggests that extended osteoid in cottoid fishes is the ideal type of system for studying the evolutionary and developmental mechanisms at work in situations of phenotypic convergence (Wake et al. 2011). Although fan‐shaped OPs appear to be ancestral with respect to perciformes and cottoids, the fork shape has emerged repeatedly, both in various sculpin clades, and outside of Cottoidei, in eelpouts. The group provides an interesting model to explore the likely possibility of evolutionary parallelism for a relatively simple trait, a phenomenon observed on a microevolutionary scale in the plate reduction of three‐spine stickleback (Cresko et al. 2004; Colosimo et al. 2005) and on a macroevolutionary scale in cases of repeated pigmentation gain or loss (Pointer and Mundy 2008; Rosenblum et al. 2010; Rogers et al. 2013). Cottoid extended osteoid also provides opportunities to identify the gene regulatory pathways involved in the mineralization of osteoid, and to investigate whether these pathways are the same across species and bones, as a similar membrane is present in the IOP and SOP of many cottoids.

### DYNAMIC DEVELOPMENT: REPLACEMENT OF MINERALIZED BONY REGIONS WITH EXTENDED OSTEOID DOES NOT OCCUR, BUT WE DO OBSERVE THE OPPOSITE

How developmentally malleable is the change in bone matrix between mineralized bone and extended osteoid? Does extended osteoid form in a region of the OP that was mineralized at an earlier stage? The flip side of this question is to ask whether a region of extended osteoid can later become mineralized. Both questions address the basic issue of developmental plasticity (and heterogeneity in form) of the bone matrix in the fork species. We looked at the distribution of extended osteoid over developmental time in multiple fork‐bearing species, assisted by superimposition of bones from different developmental stages (Fig. 6). We found no evidence that a mineralized OP region can return to a state of extended osteoid, potentially eliminating a “demineralization” mechanism from explanation. However, as the OP grows outward, regions previously occupied by extended osteoid are eventually mineralized. Successive addition of osteoid then continues at the new edge, past that of the previous stage. Whether the matrix being laid down at the growing OP edge mineralizes quickly, or persists as osteoid for an extended developmental period, depends on the species of fish and region of the bone.

We interpret this pattern to mean that extended osteoid, although present for a much longer period of time than osteoid in fan‐bearing species, is not permanently maintained during development. Maintenance of the OP shapes during juvenile development would of course be impossible without exquisite and dynamic regulation of extended osteoid—added at the growing bone edge, and at the same time overwritten by mineralization in the older matrix. Higher resolution developmental and genetic studies in the future will elucidate this dynamic developmental process, and to what extent it may vary among different lineages.

### MULTILEVEL HETEROCHRONY: FORKS REPRESENT A DERIVED, LATE‐APPEARING INSTANCE OF CELLULAR PAEDOMORPHOSIS

We observed that in both fan‐ and fork‐bearing species, the OP develops initially as a fan, but in fork‐bearing species, extended osteoid is then expressed at a later stage, presumably by blocking mineralization or by undergoing delayed mineralization. Subsequently, with continued bone growth the fan morphology changes to a fork. This temporal pattern of extended osteoid development supports a classical argument made by von Baer in the early 19th Century (von Baer 1828), and then extended by Darwin (1859), that an evolutionarily ancestral state precedes an evolutionarily derived (specialized) state in development. Based on our ASR, we conclude that this pattern is consistent with cottoid OPs: the early developmental fan state is ancestral and the following fork state possessing extended osteoid is derived.

Furthermore, considering that presence of osteoid typically marks a transient and brief early stage of bone development in vertebrates, we interpret this new pattern of delaying and lengthening the period when osteoid is prominent as indicative of heterochrony—a change in developmental timing between ancestor and descendant (Alberch et al. 1979; Klingenberg 2007). Our case seems to fit only poorly or not at all into the classical scheme of heterochrony, where changes are considered either as paedomorphic or peramorphic: In paedomorphosis, later developmental stages in the descendant retain characteristics of ancestral early stages, clearly not the case here for fork species because the ancestral early stages are fans, not forks. In peramorphosis, development in descendants is pushed beyond the end of an ancestral developmental sequence, producing a somehow exaggerated ancestral form such as the famous case of the Irish elk, possessing antlers much larger than its ancestor (Gould 1977). These definitions are, however, ill‐suited to extended osteoid and fork‐like OPs, because they refer to development strictly at the “organismal” level. Delayed mineralization, in the case of extended osteoid, occurs at the “cellular” level. Here, the extended osteoid cells (osteoblasts) retain characteristics of early (premineralization) states, but this phenomenon is nested within, and indeed occurs as a later feature of, organismal development. In other words, the derived phenotype (fork‐shaped OPs) is the result of cell‐level paedomorphosis that begins at later developmental stages in fork‐bearing species, but never initiates in fan‐bearing species. A fish with fork‐shaped OPs begins its development with osteoblasts that achieve mineralization quickly, but by the time the individual is finished developing into an adult, many of the osteoblasts composing its OPs experience a delay in their own cellular development. In this sense, the multilevel heterochronic changes in cottoid OPs have produced a form that is not at all like ancestral early or late stages, but novel. A systems‐level understanding of this particular form of novelty will require precise estimates of heterochronic shift variation among lineages with fork‐shaped OPs, in addition to molecular interrogation of the cell‐level gene regulatory processes that ultimately underlie extended osteoid.

Associate Editor: A. Goswami

## Supporting information


**Figure S1**. Nonmineralized, “extended osteoid” tissue is a prominent feature of sculpin gill cover bones.

**Figure S2**. Extended osteoid also exists in eelpouts (Zoarcidae).

**Figure S3**. Cells in extended osteoid regions express *sp7*, a transcriptional marker for osteoblasts.

**Figure S4**. OP morphology is strikingly variable across the Cottoidea radiation.

**Figure S5**. Repeatability ‐ at the species level ‐ of major OP shape metrics is high.

**Figure S6**. OP shape has evolved from an ancestral “fan” to a derived “fork” shape multiple times, largely through the parallel expansion of extended osteoid.

**Supplementary File S1**. A .xlsx file containing species information and specimen sources, for each individual fish used in the study.

**Supplementary File S2**. A .fasta file containing an alignment of assembled *sp7* DNA sequences used to design probes for *in situ* hybridization.

**Supplementary File S3**. A .xlsx file containing results from a series of phylogenetic generalized least squares (PGLS) models.

**Supplementary File S4**. A .xlsx file containing results from a series of phenotypic evolution models, fit using the *fitContinuous* function of the R package GEIGER.

Supporting Information
