# Supplementary figures and images for: Developmental tuning of mineralization drives morphological diversity of gill cover bones in sculpins and their relatives
Source: Evol Lett. 2019 Jul 16;3(4):374–91. doi: 10.1002/evl3.128 (PMC6675512; doi:10.1002/evl3.128)

**A** *Dasycottus setiger*

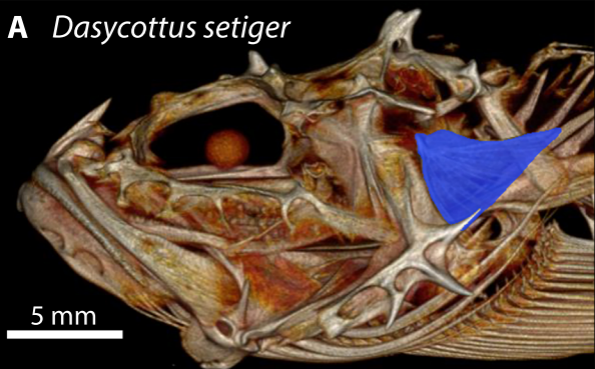

**B** *Porocottus allisi*

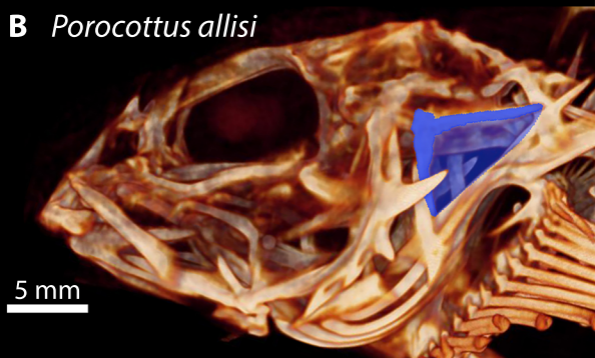

**C**

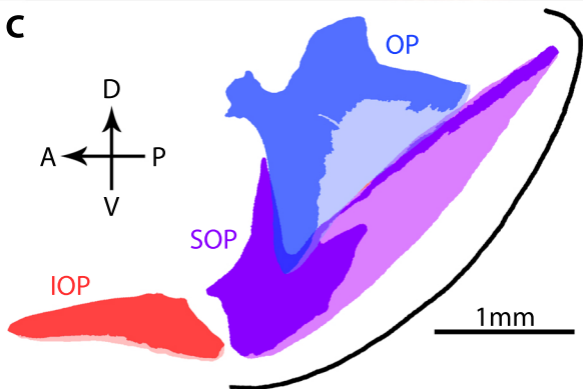

**D**

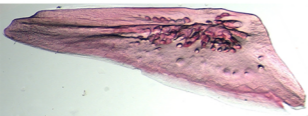

**E**

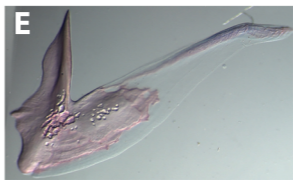

Supplement: Supplementary file 1 — Figure S1. Nonmineralized, “extended osteoid” tissue is a prominent feature of sculpin gill cover bones. [file EVL3-3-374-s001.pdf]

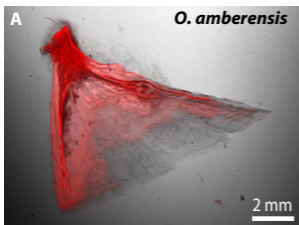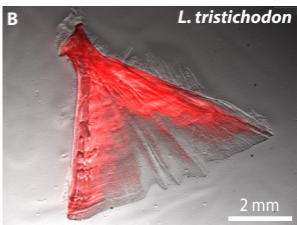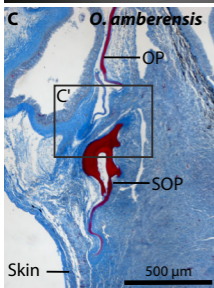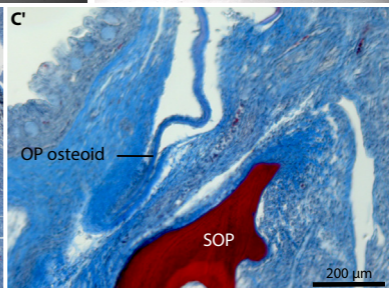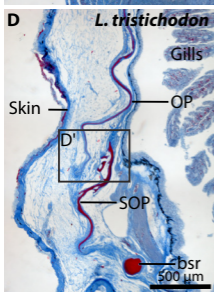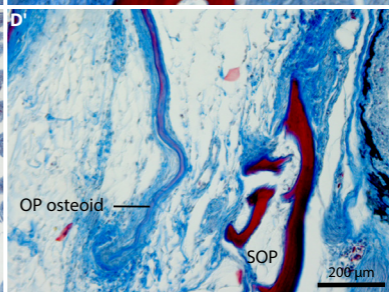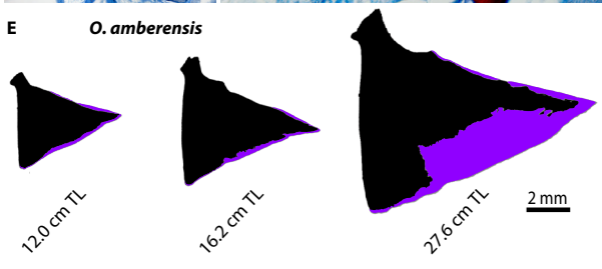

Supplement: Supplementary file 2 — Figure S2. Extended osteoid also exists in eelpouts (Zoarcidae). [file EVL3-3-374-s002.pdf]

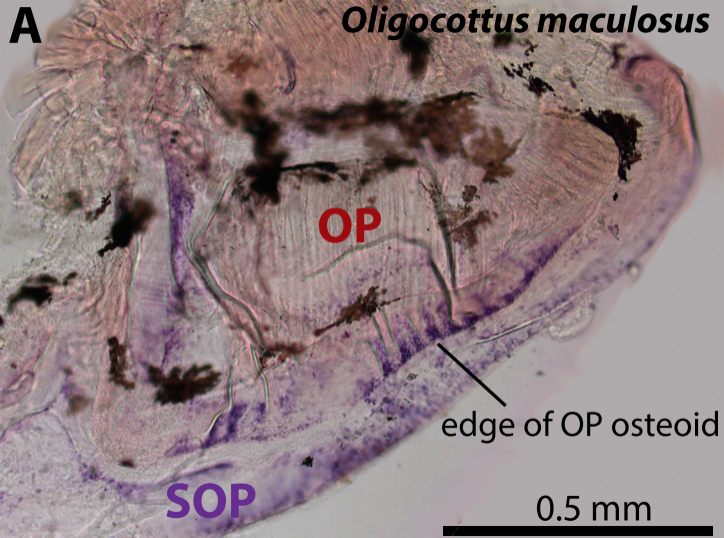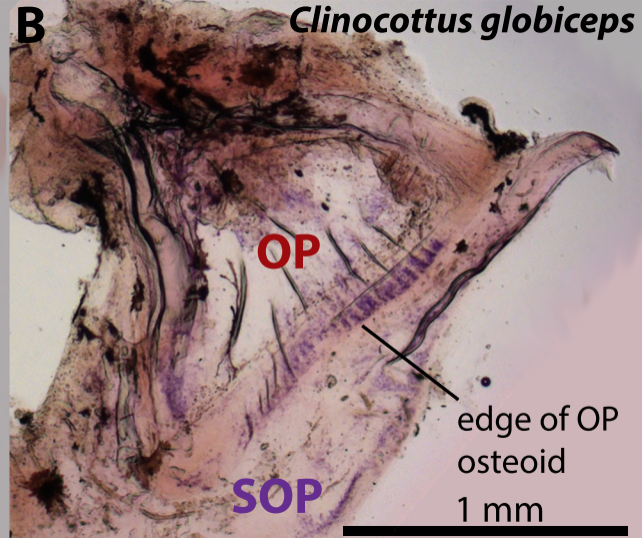

Supplement: Supplementary file 3 — Figure S3. Cells in extended osteoid regions express sp7, a transcriptional marker for osteoblasts. [file EVL3-3-374-s003.pdf]

**A****Species Replicates PCA Biplot**

PC2 (23.90% of Var. Explained)

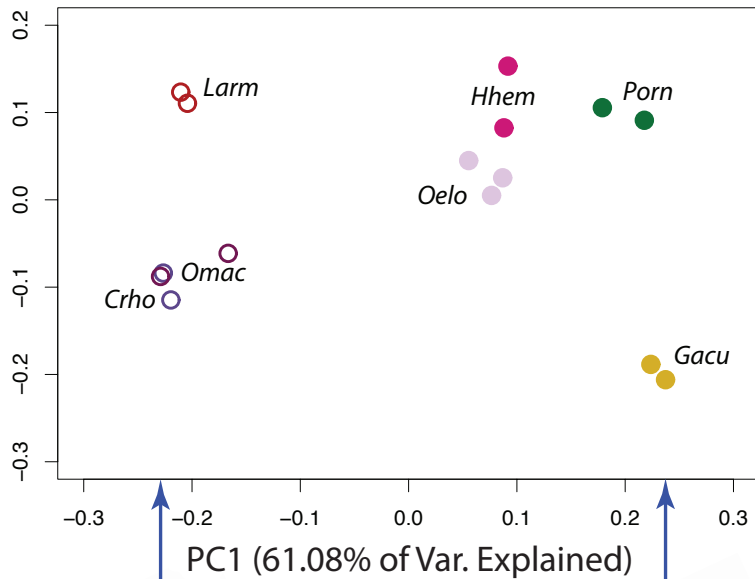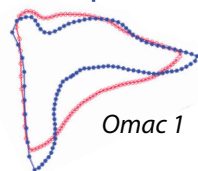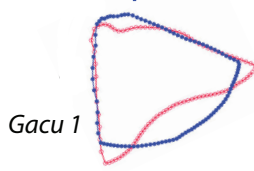**B****Within-species Repeatability (OP shape)**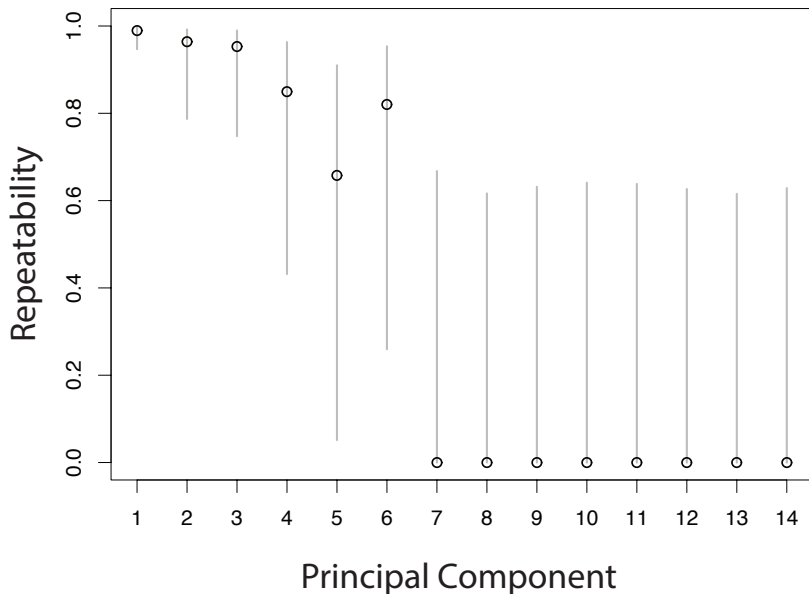

Supplement: Supplementary file 5 — Figure S5. Repeatability ‐ at the species level ‐ of major OP shape metrics is high. [file EVL3-3-374-s005.pdf]
