# Supplementary material for: Developmental tuning of mineralization drives morphological diversity of gill cover bones in sculpins and their relatives
Source: Evol Lett. 2019 Jul 16;3(4):374–91. doi: 10.1002/evl3.128 (PMC6675512; doi:10.1002/evl3.128)
Supplement: Supplementary file 4 — Figure S4. OP morphology is strikingly variable across the Cottoidea radiation. [file EVL3-3-374-s004.pdf]

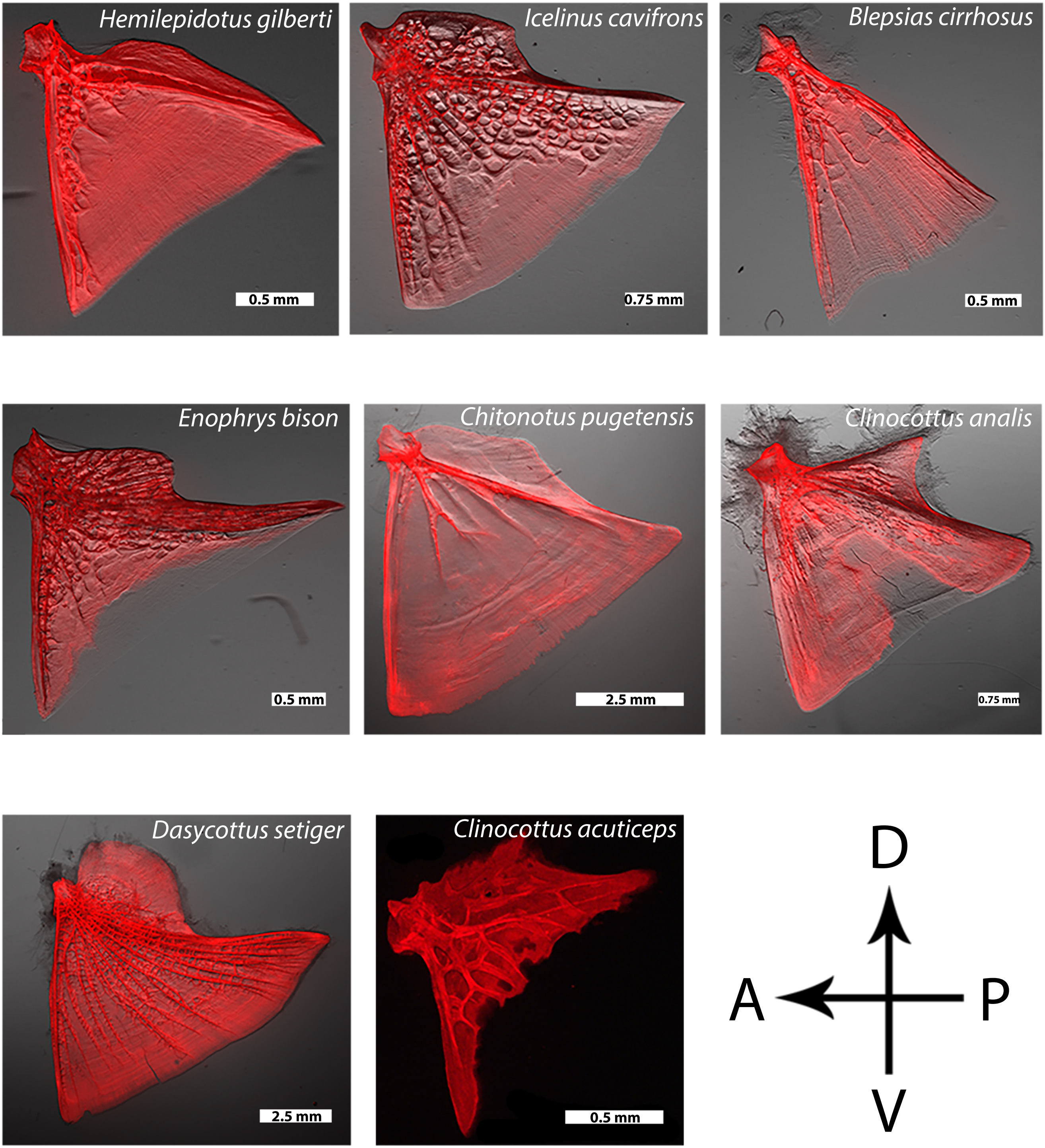

Hemilepidotus gilberti
0.5 mm
Icelinus cavifrons
0.75 mm
Blepsias cirrhosus
0.5 mm
Enophrys bison
0.5 mm
Chitonotus pugetensis
2.5 mm
Clinocottus analis
0.75 mm
Dasycottus setiger
2.5 mm
Clinocottus acuticeps
0.5 mm
D
A
P
V
