# Supplementary material for: Developmental tuning of mineralization drives morphological diversity of gill cover bones in sculpins and their relatives
Source: Evol Lett. 2019 Jul 16;3(4):374–91. doi: 10.1002/evl3.128 (PMC6675512; doi:10.1002/evl3.128)
Supplement: Supplementary file 6 — Figure S6. OP shape has evolved from an ancestral “fan” to a derived “fork” shape multiple times, largely through the parallel expansion of extended osteoid. [file EVL3-3-374-s006.pdf]

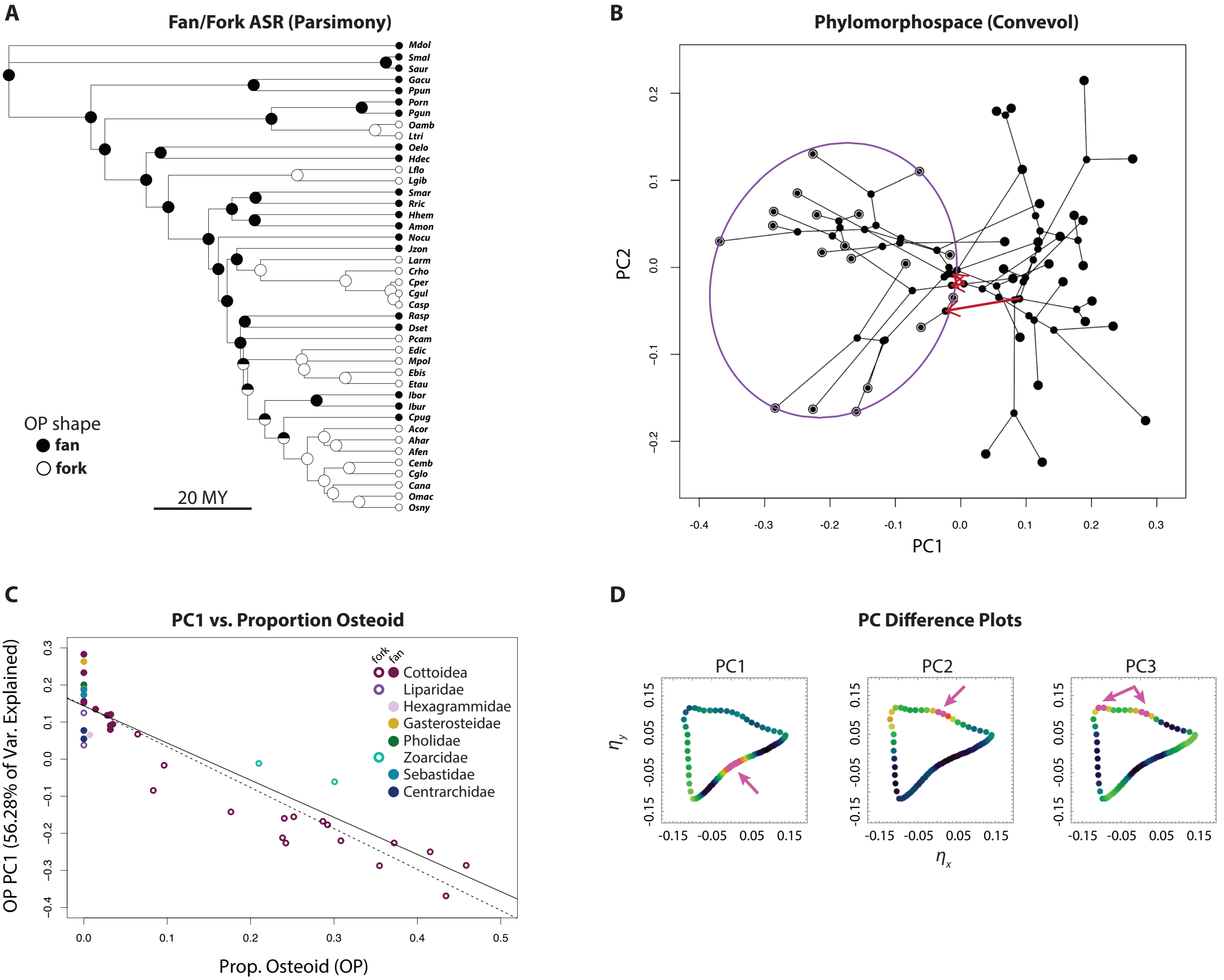
A
Fan/Fork ASR (Parsimony)
Mdol
Smal
Saur
Gacu
Ppun
Porn
Pgun
Oamb
Ltri
Oelo
Hdec
Lflo
Lgib
Smar
Rric
Hhem
Amon
Nocu
Jzon
Larm
Crho
Cper
Cgul
Casp
Rasp
Dset
Pcam
Edic
Mpol
Ebis
Etau
Ibor
Ibur
Cpug
Acor
Ahar
Afen
Cemb
Cglo
Cana
Omac
Osny
OP shape
fan
fork
20 MY
B
Phylomorphospace (Convevol)
PC2
PC1
C
PC1 vs. Proportion Osteoid
OP PC1 (56.28% of Var. Explained)
Prop. Osteoid (OP)
fork
fan
Cottoidea
Liparidae
Hexagrammidae
Gasterosteidae
Pholidae
Zoarcidae
Sebastidae
Centrarchidae
D
PC Difference Plots
PC1
PC2
PC3
η_y
η_x
