# Supplementary material for: Developmental tuning of mineralization drives morphological diversity of gill cover bones in sculpins and their relatives
Source: Evol Lett. 2019 Jul 16;3(4):374–91. doi: 10.1002/evl3.128 (PMC6675512; doi:10.1002/evl3.128)
Supplement: Supplementary file 11 — Supporting Information [file EVL3-3-374-s011.docx]

**Developmental tuning of mineralization drives morphological diversity of gill cover bones in sculpins and their relatives**

Eli G. Cytrynbaum, Clayton M. Small, Ronald Y. Kwon, Boaz Hung, Danny Kent, Yi-lin Yan, Matthew L. Knope, Ruth A. Bremiller, Thomas Desvignes, Charles B. Kimmel

**Supplementary Information**

**Supplementary Figure Legends**

**Figure S1.** Nonmineralized, “extended osteoid” tissue is a prominent feature of sculpin gill cover bones. *A*. Image from a microCT scan of the sculpin species *D*. *setiger*, in which the fully mineralized OP is indicated with a blue overlay. *B*. A similar image from a microCT scan of the sculpin species *P*. *allisi*, also with the OP indicated by a blue overlay, but in which the extended osteoid region appears as transparent in the scan. The preopercle (“POP”), which is not a focus of this study, is visible in both images as the large spinose bone just anterior and ventral to the OP, and is likely involved in predator defense. Original images are from the Open Science Framework’s “Scan All Fishes” project, were contributed by S. C. Farina (2015) and T. J. Buser and A. P. Summers (2016), and may be found at <https://osf.io/uqg4j/> and <https://osf.io/5kdxw/>, respectively. *C*. A simplified cartoon of three major gill cover bones, drawn from the “fork-bearing” *Oligocottus maculosus*. The opercle (OP) in blue, the subopercle (SOP) in purple, and the interopercle (IOP) in red are drawn to emphasize mineralized (dark) and osteoid (light) regions. *D*-*E*. Actual images of an *O*. *maculosus* IOP and SOP, respectively, with mineralized bone stained pink by Alizarin Red and extended osteoid shown by the transparent, unstained regions. (See Fig. 1 for an Alizarin Red-stained OP image of this species.)

**Figure S2.** Extended osteoid also exists in eelpouts (Zoarcidae). Adult *Ophthalmolycus amberensis* (*A*.) and *Lycenchelys tristichodon* (*B*.) OPs stained with Alizarin Red, showing lack of stain in the region of extended osteoid. Cross sections of Ralis-Watkins-stained *O. amberensis* (*C*.-*C*.*'*) and *L*. *tristichodon* (*D*.-*D*.*'*) gill covers, with the mineralized portions of the OP and SOP in red and the extended osteoid in blue, showing a lack of mineralization in the extended osteoid region. bsr = branchiostegal ray. *E*. In *O. amberensis*, extended osteoid and the resulting "fork" morphology appear after an established "fan" shape, relatively late in OP development. TL = total length.

**Figure S3.** Cells in extended osteoid regions express *sp7*, a transcriptional marker for osteoblasts. *A*. A juvenile *Oligocottus maculosus* OP and SOP. Deep purple indicates *in situ* hybridization signal for *sp7*, expressing most strongly along the growing edges of the extended osteoid on the posterior ventral edges of the OP and SOP, but also to a lesser extent in non-extended osteoid regions. *B*. Same image for a juvenile *Clinocottus globiceps*.

**Figure S4.** OP morphology is strikingly variable across the Cottoidea radiation. Note the variation in this phylogenetically diverse panel of Alizarin Red-stained adult OPs and the clear “growth bands” continuous between extended osteoid and mineralized tissues in *E. bison* and *C. analis*. Not only do the bones differ with respect to extended osteoid, but they are highly variable regarding general 2-D shape, thinness, presence of “trabeculae” (e.g. *I. cavifrons* and *E. bison*) and presence of ridges (e.g. *D. setiger*).

**Figure S5.** Repeatability - at the species level - of major OP shape metrics is high. *A*. First two Principal Components (PCs) from an OP eigenshape analysis based only on those species for which two or more individual animals were available. In morphospace defined by PC1 and PC2, individuals from the same species resemble one another more, on average, than they do individuals from different species. Wireframes representing shape variants at low and high PC1 values (in blue) illustrate that PC1 largely separates “fork-” from “fan-bearing” species. *B.* Species-level repeatability estimates and 95% confidence intervals (see Methods) for all 14 PCs from the same eigenshape analysis. For the first three PCs, within-species variation is minimal relative to among-species variation, as indicated by high repeatability.

**Figure S6.** OP shape has evolved from an ancestral “fan” to a derived “fork” shape multiple times, largely through the parallel expansion of extended osteoid. *A*. Parsimony-based ancestral state reconstructions (see Methods) for the binary trait of fan-/fork-shaped OP. Equally parimonius ancestral states are indicated by half-filled circles and represent regions of especially high uncertainty. Because an unrooted tree (with a single outgroup taxon) was required for this analysis, please note that the branch leading to Mdol is shorter than the actual Mdol:ingroup diverence time. *B*. Phylomorphospace defined by PC1 and PC2 from OP eigenshape analysis, and produced from the *convevol* analysis (see Methods). Large circles are terminal nodes (species) and small circles represent internal (ancestral) nodes. The presumed region of convergence, defined by clearly fork-like OPs with extended osteoid, is shown here as a purple ellipse. The three red arrows, which are clearly in the fan-to-fork direction along PC1, show the number of independent “invasions” of the zone of convergence, and correspond to C_5_ = 3. *C*. Scatterplot showing the strong negative relationship between PC1 values and the proportion of the OP composed of extended osteoid. Taxonomic group colors are the same as in Fig 3. The solid line represents the slope and intercept from ordinary least squares regression, and the dashed line represents these parameters from phylogenetic generalized least squares regression. *D*. “Difference plots” for PCs 1-3, which permit visualization of shape variation “hot spots” in the semi-landmark space (*η_y_* vs. *η_x_*) via points that represent semi-landmark coordinate averages. The “hottest” colors (i.e. pink, indicated by arrows) denote outline regions of the greatest shape variation. PC1, accounting for over half of the variance, contributes heavily to diversity in the region that defines the fan-fork continuum. PCs 2 and 3 explain variation independent of the region affected by osteoid, but in regions likely to affect muscle attachment (see Discussion).

**Supplementary File Legends**

**Supplementary File 1.** A .xlsx file containing species information and specimen sources, for each individual fish used in the study. OCA = Oregon Coast Aquarium, OS = Oregon State University, UW = University of Washington Burke Museum, VA = Vancouver Aquarium. Museum collection numbers are indicated when available.

**Supplementary File 2.** A .fasta file containing an alignment of assembled *sp7* DNA sequences used to design probes for *in situ* hybridization. Included are protein-coding sequences from *Cottus perplexus*, *Clinocottus globiceps*, and *Oligocottus maculosus*.

**Supplementary File 3.** A .xlsx file containing results from a series of phylogenetic generalized least squares (PGLS) models. Three major “sets” of models were fit to the data, and within-set comparisons were accomplished via AICc and/or likelihood ratio test (LRT). The first set evaluated variation in PC1 as a function of two habitat states (“deep” and “shallow”) and OP size. The second set evaluated variation in PC1 as a function of three habitat states (“deep marine,” “shallow marine,” and “freshwater/estuary”) and OP size. The third set evaluated variation in the proportion of OP area composed of extended osteoid as a function of the aforementioned two habitat states and OP size. For each set, one full and two reduced models (each reduced model obtained by dropping either explanatory variable) were fit to the data, assuming each of three evolutionary models: Brownian motion (BM), Pagel’s lambda (PagLamb), and Ornstein-Uhlenbeck (OU). Estimates for lambda are given in the case of PagLamb. Minimum AICc values (and those < 2 units above the minimum) for each set are highlighted in green. In cases of comparison bewteen full and reduced models, the likelihood ratio test statistic (LR) is given, along with the degrees of freedom and the *p*-value for the LRT. LRT *p*-values < 0.05 are highlighted in red.

**Supplementary File 4.** A .xlsx file containing results from a series of phenotypic evolution models, fit using the *fitContinuous* function of the R package GEIGER. Four models each for the two correlated traits PC1 and proportion of OP composed of osteoid were fit and compared separately using AICc. Minimum AICc values (and those < 2 units above the minimum) for each trait are highlighted in green.
